# Supplementary figures and images for: Risk factors for predicting mortality of COVID-19 patients: A systematic review and meta-analysis
Source: PLoS One. 2020 Nov 30;15(11):e0243124. doi: 10.1371/journal.pone.0243124 (PMC7703957; doi:10.1371/journal.pone.0243124)

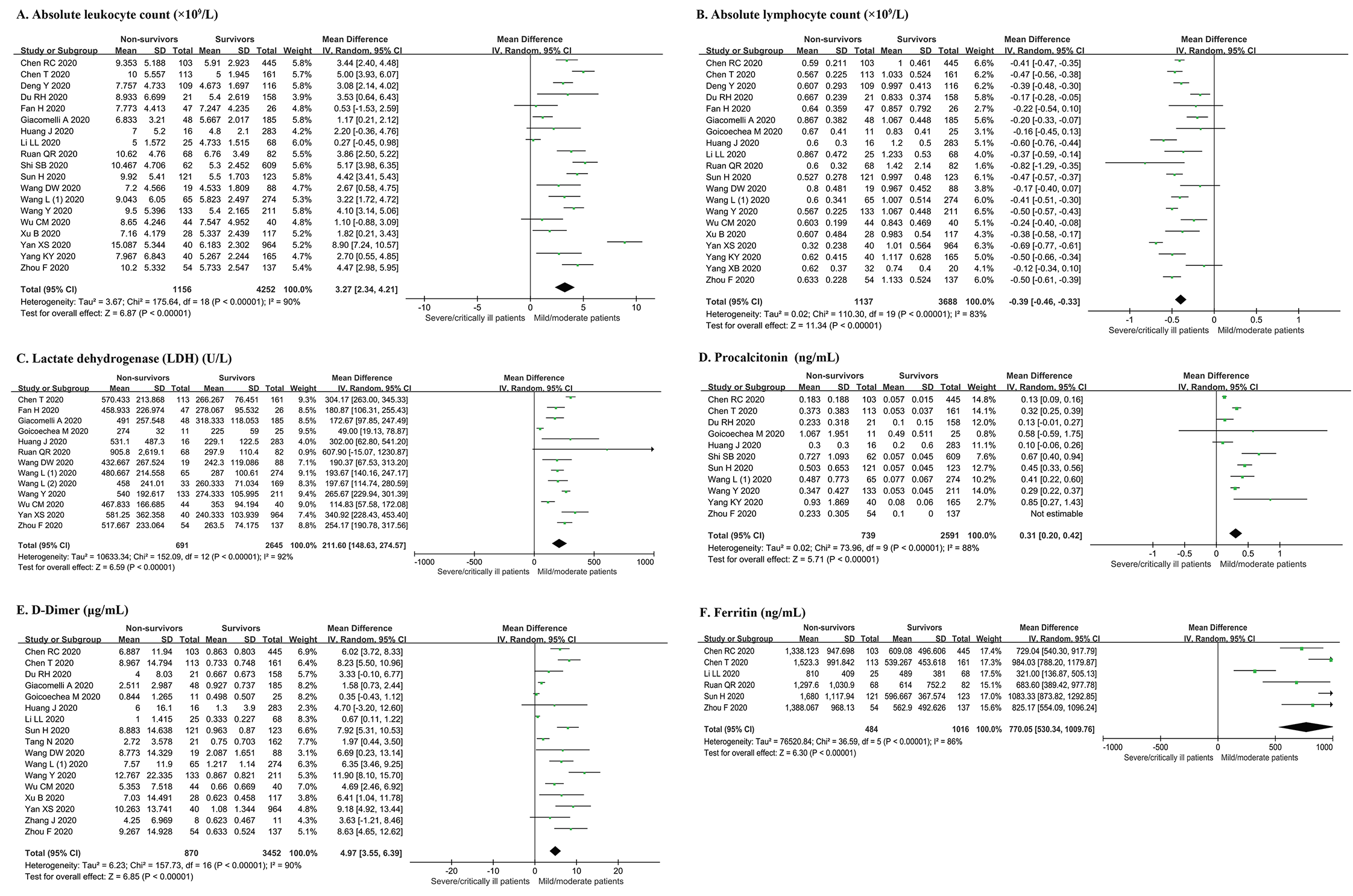

Supplement: S1 Fig — Forest plot of the laboratory abnormalities (A) leukocytes, (B) lymphocytes, (C) lactate dehydrogenase (LDH), (D) procalcitonin, (E) D-Dimer, (F) ferritin levels in survivors versus non-survivors. (TIF) [file pone.0243124.s001.tif]

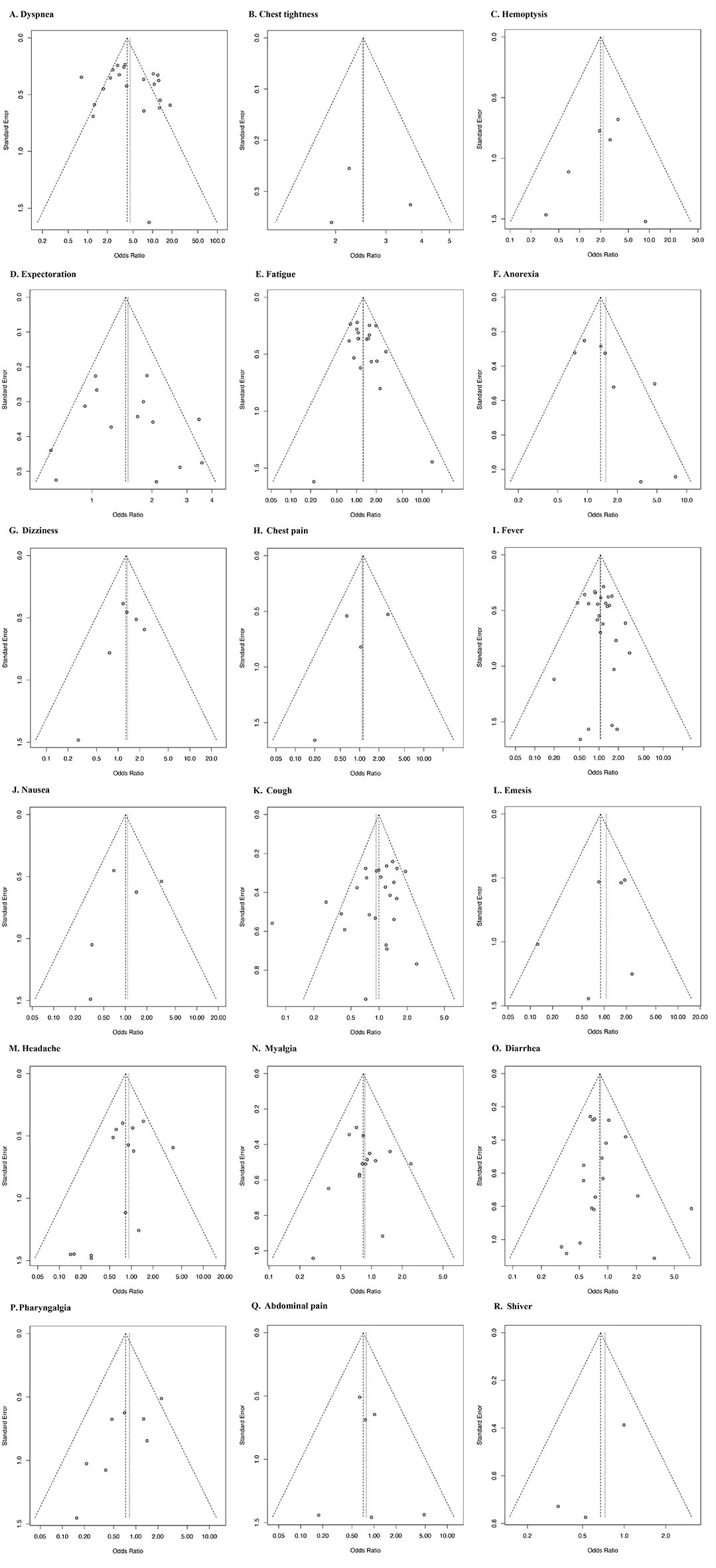

Supplement: S2 Fig — The publication bias of the clinical characteristics (A. dyspnea; B. chest tightness; C. hemoptysis; D. expectoration; E. fatigue; F. anorexia; G. dizziness; H. chest pain; I. fever; J. nausea; K. cough; L. emesis; M. headache; N. myalgia; O. diarrhea; P. pharyngalgia; Q. abdominal pain; R. shiver) between survivors and non-survivors. (TIF) [file pone.0243124.s002.tif]

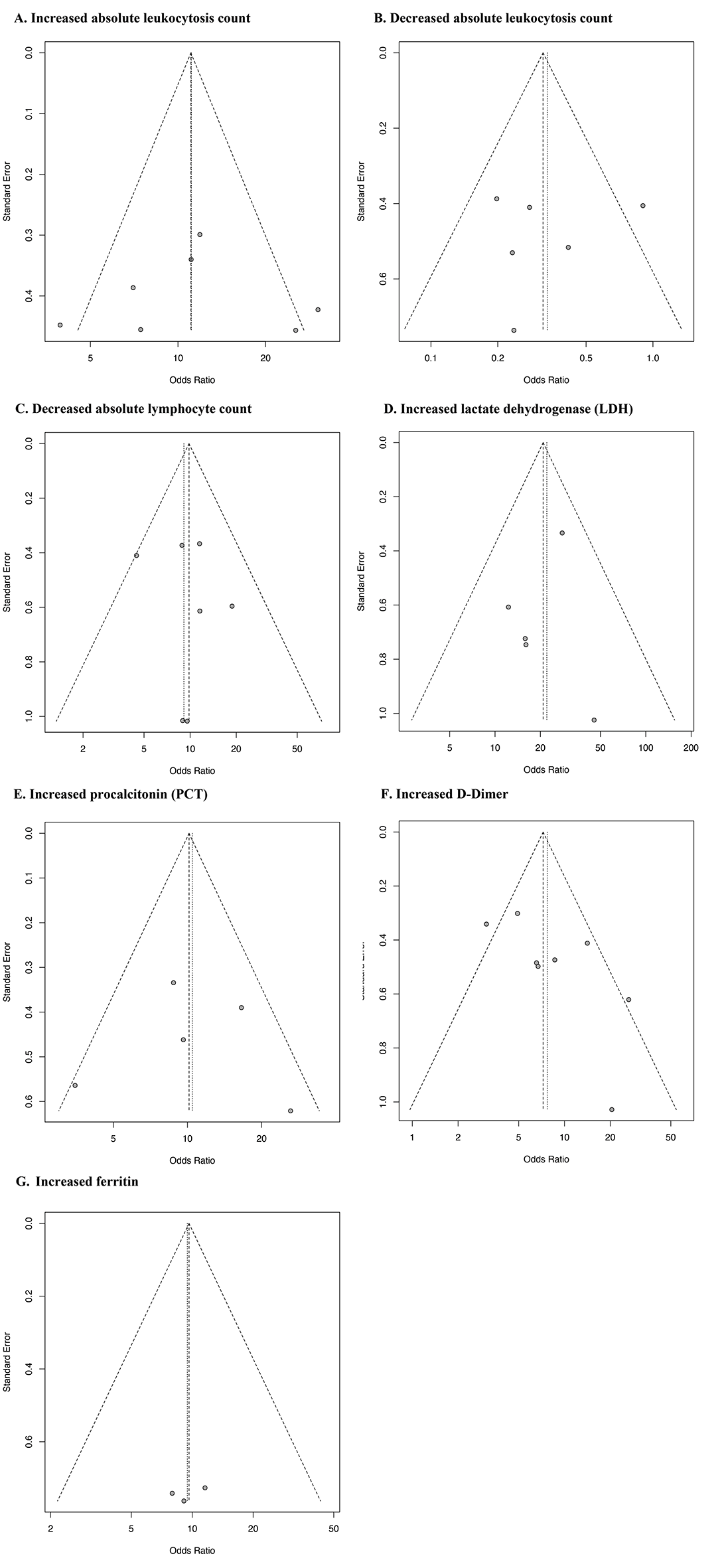

Supplement: S3 Fig — The publication bias of the laboratory abnormalities (A) increased leukocytes, (B) decreased leukocytes, (C) decreased lymphocytes, (D) increased lactate dehydrogenase (LDH), (E) increased procalcitonin (PCT), (F) increased D-Dimer, (G) increased ferritin between survivors and non-survivors. (TIF) [file pone.0243124.s003.tif]
