# Supplementary material for: Risk factors for predicting mortality of COVID-19 patients: A systematic review and meta-analysis
Source: PLoS One. 2020 Nov 30;15(11):e0243124. doi: 10.1371/journal.pone.0243124 (PMC7703957; doi:10.1371/journal.pone.0243124)
Supplement: S2 File — (DOCX) [file pone.0243124.s008.docx]

**S2 File**

**Search strategy for meta-analysis of Clinical characteristics and laboratory abnormalities of COVID-19 patients with and without severe disease (PubMed via NLM)**

#1 Search ((((2019 novel coronavirus disease) OR severe acute respiratory syndrome coronavirus 2) OR COVID-19) OR 2019-nCoV) OR SARS-CoV-2

#2 Search ((((((Mortality) OR fatality) OR fatal) OR mortal) OR death) OR Lethality)

#3 Search ((((Clinical) OR Chemistry) OR Laboratory) OR Risk factor)

#4 Search #1AND #2 AND #3 Filters: in the last 1 year
