# Supplementary material for: Risk factors for predicting mortality of COVID-19 patients: A systematic review and meta-analysis
Source: PLoS One. 2020 Nov 30;15(11):e0243124. doi: 10.1371/journal.pone.0243124 (PMC7703957; doi:10.1371/journal.pone.0243124)
Supplement: Attachment — Submitted filename: Response to Reviewers.docx [file pone.0243124.s009.docx]

Dear Editor-in-Chief and reviewers:

Thank you for your letter and for the reviewers' comments concerning our manuscript entitled “Risk factors for predicting mortality of COVID-19 patients: A systematic review and meta-analysis”. All suggestions were very helpful for us to revise and improve our paper. We carefully studied these comments and made corrections that we hope meet with approval. The revised portions are marked with ‘Track changes’ in the manuscript.

**Here are my responses to the Editor-in-Chief’ comments.**

1. Please ensure that your manuscript meets PLOS ONE's style requirements, including those for file naming. The PLOS ONE style templates can be found at

https://journals.plos.org/plosone/s/file?id=wjVg/PLOSOne_formatting_sample_main_body.pdf and

https://journals.plos.org/plosone/s/file?id=ba62/PLOSOne_formatting_sample_title_authors_affiliations.pdf

Response: We have now re-formatted our paper carefully to meet PLOS ONE’s style requirements.

2. Please attach a Supplemental file of the results of the quality assessment for each individual study assessed, reporting the outcome for each individual criteria considered.

Response: The results of the quality assessment for each individual study were presented in S1 Table. We have added S1 table in the revised version of our manuscript. We change the original “eTable 1” to “S2 Table” and the original “eTable 2” to “S3 Table”. We are sorry for making some mistakes in calculating the MINORS scores. After re-calculating all scores of enrolled studies, the MINORS score of Chen T(2020) was changed from 18 to 21, the MINORS score of Goicoechea M (2020) was changed from 21 to 18, and the MINORS score of Zhou F (2020) was changed from 18 to 21.(Page 8-10, Table 1)

3. Please include the date(s) on which you accessed the databases or records to obtain the data used in your study.

Response: We conducted a systematic search in PubMed, Scopus, Web of Science and Embase to identify studies in patients with COVID-19 infection up to 4 June 2020. This was mentioned in “Materials and methods”-“Search strategy”. (Page 4, Line 64).

4. Please provide a citation for the MINORS score.

Response: The citation for the MINORS score was provided as reference [3]. (Page 5, Line 88)

Slim K, Nini E, Forestier D, Kwiatkowski F, Panis Y, Chipponi J. Methodological index for non-randomized studies (MINORS): development and validation of a new instrument. ANZ Journal of Surgery. 2003;73(9):712-6.

5.Thank you for stating the following in the Funding Section of your manuscript:

[This work was supported by National Nature Science Foundation of China [grant

227 numbers 91859203 and 81871890] and Major Science and Technology Innovation

228 Project of Chengdu City [grant number 2020-YF08-00080-GX]]

We note that you have provided funding information that is not currently declared in your Funding Statement. However, funding information should not appear in the Acknowledgments section or other areas of your manuscript. We will only publish funding information present in the Funding Statement section of the online submission form.

Please remove any funding-related text from the manuscript and let us know how you would like to update your Funding Statement. Currently, your Funding Statement reads as follows:

[The author(s) received no specific funding for this work.]

Please include your amended statements within your cover letter; we will change the online submission form on your behalf.

Response: We have removed any funding-related text from the manuscript and add the information of funding in cover letter.

6. We suggest you thoroughly copyedit your manuscript for language usage, spelling, and grammar. If you do not know anyone who can help you do this, you may wish to consider employing a professional scientific editing service.

Response: The full manuscript has been reviewed and edited by a professional scientific English editor.

**Here are my responses to the** **reviewers’ comments.**

Reviewer #1: The authors aimed to perform a systematic meta-analysis to summarize the clinical characteristics and laboratory test before treatment among COVID-19 patients 65 and identify the possible risk factors for mortality. The article is timely and novel and it is overall well structured and written.

Nevertheless, I would improve the manuscript focusing also on cardiovascular disease that increases poor prognosis and related mortality. For this purpose read and cite the article by Ielapi N, et al. Cardiovascular disease as a biomarker for an increased risk of COVID-19 infection and related poor prognosis. Biomark Med. 2020 Jun;14(9):713-716.

Response: Thank you for reviewing our manuscript and your advices were helpful. According to your suggestion about the impact of cardiovascular disease on COVID-19 infection and prognosis. We did analysis to detect the relationship between hypertension and chronic cardiac disease and mortality of COVID-19. The results showed that hypertension (OR= 2.94, 95%CI [2.39, 3.62], P<0.001) and chronic cardiac disease (OR= 3.89, 95%CI [2.65, 5.72], P<0.001), were also associated with increased mortality of COVID-19. The detail information was provided in the following table.

|  | No. of the studies | No. of the patients | OR, 95%CI | P-value | Heterogeneity | |
| --- | --- | --- | --- | --- | --- | --- |
|  |  |  |  |  | I^2^ | P-value |
| Hypertension | 28 | 8939 | 2.94 [2.39, 3.62] | <0.001 | 54.90% | <0.001 |
| CCD | 17 | 3806 | 3.89 [2.65, 5.72] | <0.001 | 53.40% | 0.005 |

We appreciate the editor/reviewers' earnest work and hope that the corrections will make the revised manuscript acceptable for publication. Once again, thank you very much for your comments and suggestions, and we look forward to hearing from you.
